# Compensatory rearrangement of parvalbumin interneuron voltage‐gated sodium channel subunits in a mouse model of Dravet syndrome

**DOI:** 10.1002/epi.70336

**Published:** 2026-06-08

**Authors:** Ania K. Dabrowski, Ala Somarowthu, Sophie R. Liebergall, Damaris N. Lorenzo, Ethan M. Goldberg

**Affiliations:** ^1^ Division of Neurology, Department of Pediatrics Children's Hospital of Philadelphia Philadelphia Pennsylvania USA; ^2^ Department of Neuroscience Perelman School of Medicine at University of Pennsylvania Philadelphia Pennsylvania USA; ^3^ Department of Cell and Developmental Biology University of Pennsylvania Philadelphia Pennsylvania USA; ^4^ Department of Physiology Perelman School of Medicine at University of Pennsylvania Philadelphia Pennsylvania USA; ^5^ Epilepsy NeuroGenetics Initiative Children's Hospital of Philadelphia Philadelphia Pennsylvania USA; ^6^ Department of Neurology Perelman School of Medicine at University of Pennsylvania Philadelphia Pennsylvania USA; ^7^ Present address: Division of Neurology, Stead Family Department of Pediatrics, Carver College of Medicine University of Iowa Iowa City Iowa USA

**Keywords:** axon initial segment, Dravet syndrome, Na_V_1.1, Na_V_1.6, *SCN1A*, voltage‐gated sodium channels

## Abstract

Heterozygous loss‐of‐function variants in the gene *SCN1A*, which encodes the voltage‐gated sodium channel (VGSC) pore‐forming (α) subunit Na_V_1.1, lead to a spectrum of neurological disease, including Dravet syndrome. Na_V_1.1 is prominently expressed at the proximal portion of the axon initial segment (AIS) of fast‐spiking γ‐aminobutyric acidergic parvalbumin‐expressing inhibitory interneurons (PV+INs). In Dravet syndrome (*Scn1a* ^*+/−*^ haploinsufficient) mice, action potential firing is impaired in PV+INs during postnatal development; however, in mature animals, PV+IN fast‐firing frequency has recovered. We used detailed immunohistochemistry and microscopy to probe the mechanism of this functional recovery, investigating potential upregulation of other brain‐expressed VGSC subunits at the PV+IN AIS. We found a specific upregulation of Na_V_1.6 immunofluorescence at the AIS of PV+INs in adult (but not developing) *Scn1a* ^*+/−*^ mice compared to wild type, with no upregulation of other brain‐expressed subunits Na_V_1.2 or Na_V_1.3. These results demonstrate one mechanism through which epilepsy‐related gene variants reorganize the developing brain and enact endogenous changes that may be at least partially compensatory.

**Key points**

- Dravet syndrome is due to heterozygous pathogenic loss‐of‐function variants in *SCN1A* encoding the sodium channel subunit Na_V_1.1.
- Loss of Na_V_1.1 leads to transient impairment of action potential generation in PV+INs from developing *Scn1a* ^+/−^ mice that recovers by young adulthood.
- Recovery of action potential generation is correlated with selective upregulation of Na_V_1.6 at the PV+IN axon initial segment in young adulthood.
- This finding may explain the mechanistic basis of recovery of PV+IN action potential generation with development.
- However, the observed upregulation of Na_V_1.6 may not be sufficient to recover impaired PV+IN synaptic transmission.

## 1 INTRODUCTION

Information transfer in the brain requires precise regulation of electrical activity, the basis of which is complex patterns of action potentials (APs). APs are generated at the axon initial segment (AIS), a highly specialized subcellular compartment that contains a dense, cell type‐specific array of ion channels, cytoskeletal proteins, and signaling molecules.^1^ Perturbation of AIS structure and function leads to neurological disease, including epilepsy.

Na_V_1.1 is an AIS‐localized voltage‐gated sodium channel (VGSC) encoded by the *SCN1A* gene. Variants in *SCN1A* cause a spectrum of human disease, including most prominently Dravet syndrome (DS), a developmental and epileptic encephalopathy, defined by treatment‐resistant epilepsy, temperature sensitivity, features of or formal diagnosis of autism spectrum disorder, and sudden unexplained death in epilepsy (SUDEP).^2^ In mice, *Scn1a* haploinsufficiency (*Scn1a* ^*+/−*^) closely models many (but perhaps not all) core DS phenotypes, with spontaneous and temperature‐sensitive seizures, behavioral abnormalities, and increased seizure‐related mortality.^3^, ^4^ Na_V_1.1 is critical for generation and propagation of APs in γ‐aminobutyric acidergic (GABAergic) interneurons, particularly parvalbumin‐expressing interneurons (PV+INs), where Na_V_1.1 is localized to the proximal AIS (nearest the soma) and nodes of Ranvier.^5^, ^6^ Decreased Na_V_1.1 expression leads to impaired PV+IN fast‐spiking properties in developing mice. However, PV+IN firing frequency has recovered to nearly wild‐type (WT) levels in juvenile *Scn1a* ^*+/−*^ mice by postnatal day (P) 35.^7^ Yet, the mechanism underlying this functional recovery is unknown.

Na_V_1.1 is one of four VGSC α subunits expressed in the central nervous system (CNS), together with Na_V_1.2, Na_V_1.3, and Na_V_1.6, each with unique developmental, cell type‐specific, and subcellular expression.^8^, ^9^ Na_V_1.3 predominates during embryogenesis but declines postnatally, when it is replaced by other VGSCs. Na_V_1.1 and Na_V_1.2 are expressed in largely mutually exclusive neuronal subtypes; both are initially localized to the proximal AIS, although Na_V_1.2 shifts dendritically with development. Na_V_1.6 is distal to Na_V_1.1 or Na_V_1.2 in most neurons, including PV+INs.

In this study, we tested whether recovery of fast‐spiking properties of PV+INs in *Scn1a* ^*+/−*^ mice by young adulthood might involve compensatory upregulation of CNS‐expressed VGSC α subunits, either through increased Na_V_1.1 protein levels despite genetic haploinsufficiency or through upregulation of a different subunit.

## 2 MATERIALS AND METHODS

### 2.1 Mice

Experiments were conducted in accordance with National Institutes of Health guidelines and with approval of the institutional animal care and use committee of the Children's Hospital of Philadelphia. The following mouse strains were used: *Scn1a* ^*+/−*^ mice (RRID:MMRRC_037107-JAX) were maintained on a strict 50:50129S6:C57BL6/J background (RRID:IMSR_TAC:129sve and RRID:IMSR_JAX:000664) as previously described.^7^, ^10^

#### 2.1.1 Immunohistochemistry

Animals were anesthetized with isoflurane and perfused with phosphate‐buffered saline then ice cold glyoxal solution (9% glyoxal, 8% acetic acid, pH = 4).^11^ Brains were removed and postfixed overnight in glyoxal solution, then processed through sucrose gradients, mounted in Tissue‐Tek(R) O.C.T. (Optimal Cutting Temperature) compound, frozen, and sectioned (50 μm). Sections were stained with primary antibodies (all 1:300 unless otherwise noted) Na_V_1.1 (IgG1, Neuromab 75–023, RRID:AB_2238842), Na_V_1.2 (IgG2A, NeuroMab 75–024, RRID:AB_2184030), Na_V_1.3 (rabbit, Millipore AB5208, RRID:AB_11212648), Na_V_1.6 (IgG1, NeuroMab 75–026, AB_2184197), parvalbumin (rabbit, Swant PV27, RRID:AB_2631173, 1:1000), parvalbumin (IgG1, Millipore MAB1572, RRID:AB_2174013), and ankyrin‐G (AnkG; guinea pig, Synaptic Systems 386005, RRID:AB_2737033) and secondary antibodies (all Alexa Fluor conjugates, Molecular Probes, 1:500) anti‐IgG1 AF488 (A21121, RRID:AB_2535764), anti‐IgG2a AF488 (A21131, RRID:AB_141618), antirabbit AF488 (A11034, RRID:AB_2576217), anti‐IgG1 AF568 (A21124, RRID:AB_141611), antirabbit AF568 (A11036, RRID:AB_10563566), and anti‐guinea pig AF633 (A21105, RRID:AB_2535757).

### 2.2 Image acquisition

The PV+IN AIS was imaged in somatosensory cortex layers 2/3 to layer 5. The AIS was identified by AnkG staining^12^ that was colocalized with parvalbumin‐expressing cells. Images were acquired via confocal microscopy (Leica SP8) at 40× with (Hybrid Detection) detection and 4× zoom. To increase signal‐to‐noise during acquisition, Na_V_ channel signal (*n* = 488) were acquired with 3× line accumulation in LAS X software and AnkG channel signal (*n* = 633) with 2× line accumulation.

### 2.3 Image analysis

Na_V_ intensity was measured in three dimensions along the AIS as defined by PV+IN‐associated AnkG staining. A three‐dimensional cylindrical mask was traced from proximal location at the soma distally along the AnkG signal using the Fiji NeuroSNT plug‐in.^13^, ^14^ Because background fluorescence intensity varied with z‐plane in each image, Na_V_ absolute intensity was normalized to the mean background intensity of the entire corresponding z‐plane. Normalized Na_V_ subunit intensity was binned into .5‐μm bins along the AIS.

### 2.4 Statistical analysis

Data were analyzed using linear mixed‐effects models implemented in MATLAB (fitlme). VGSC intensity was modeled as a function of AIS proximal–distal localization, genotype, Na_V_1.X subunit, and age as fixed effects, including all interaction terms. A random‐intercept structure was specified for mouse and cell nested within mouse, with random slopes included for selected within‐group factors. Models were fit using restricted maximum likelihood, and categorical predictors were coded using effects coding. An isotropic covariance structure was assumed for the random effects. Model fit was assessed using the coefficient of determination (*R* ^2^). Fixed effects were evaluated using type III analysis of variance (ANOVA) with Satterthwaite approximation for degrees of freedom.

To further investigate interaction effects, post hoc analyses were conducted separately within each Na_V_1.X subunit and subgroup. For each combination of Na_V_1.X subunit and age group, a reduced mixed‐effects model was fit including genotype and AIS localization as fixed effects, with their interaction. The effect of genotype was assessed using ANOVA. Similarly, for each combination of Na_V_1.X subunit and genotype, a reduced model including age group and AIS localization was fit, and the effect of age group was evaluated. All models retained the same random‐effects structure as the full model. Multiple comparisons across post hoc tests were controlled using Bonferroni correction.

## 3 RESULTS

**FIGURE 1:**
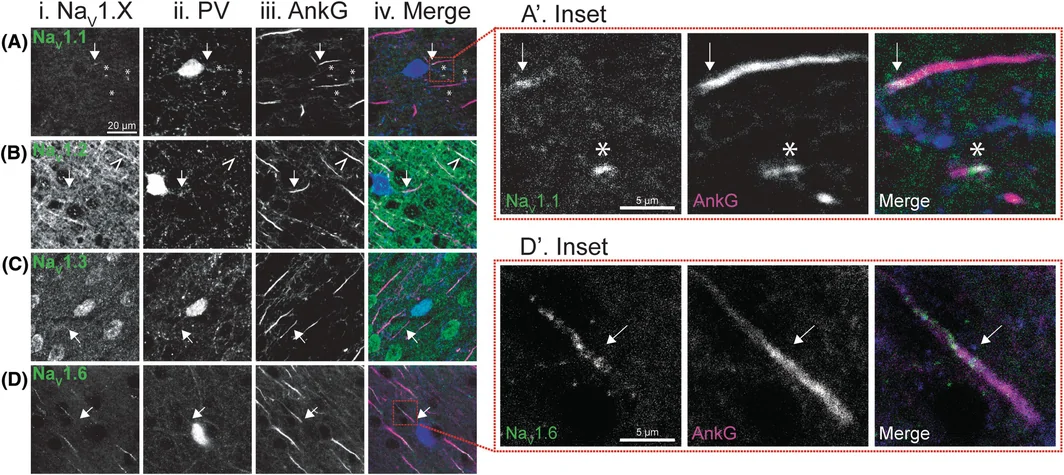
Voltage‐gated sodium channel subunit expression in wild‐type somatosensory neocortex at postnatal day. (A) Na_V_1.1 is expressed at the proximal segment of the PV+IN AIS (arrows) and apparent nodes of Ranvier (asterisks). (B) Na_V_1.2 is expressed in neuropil and the axon initial segment (AIS) of distributed neurons but is not present at the parvalbumin‐expressing interneuron (PV+IN) AIS (arrows). (C) Na_V_1.3 is expressed at the soma (but not clearly at the membrane) but not at the PV+IN AIS (arrows). (D) Na_V_1.6 is expressed at the distal AIS of both PV+INs (arrow) and non‐PV+INs. Each image is a summed projection of 20 sections. Higher magnification images of the area indicated within dashed red squares are shown at in the insets at right. Na_V_ subunits in green; parvalbumin (PV) in blue; ankyrin‐G (AnkG) in magenta. Scale bars = 20 μm (left) and 5 μm (insets).

**FIGURE 2:**
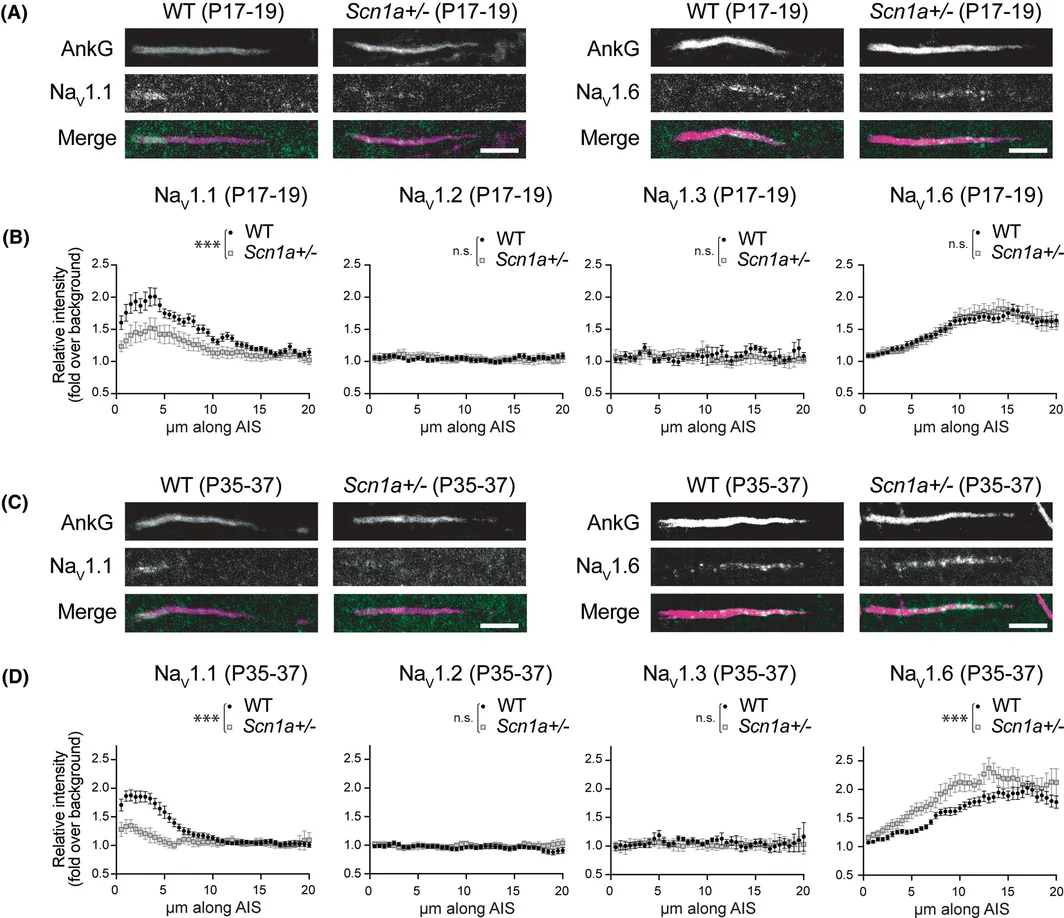
Voltage‐gated sodium channel (VGSC) subunit quantification at parvalbumin‐expressing interneuron (PV+IN) axon initial segments (AISs) in wild‐type (WT) and *Scn1a* ^*+/−*^ at postnatal day (P) 17–P19 and P35–P37. (A, C) Representative images of Na_V_1.1 and Na_V_1.6 at P17–P19 (A) and P35–P37 (C) in WT and *Scn1a* ^*+/−*^ PV+INs. Na_V_ is in green, AnkG in magenta. Scale bars = 5 μm. (B, D) Quantification of Na_V_1.1, Na_V_1.2, Na_V_1.3, and Na_V_1.6 intensity at P17–P19 and P35–P37 as intensity normalized to mean background. The x‐axis represents distance from soma along the ankyrin‐G (AnkG)‐positive AIS. Each point represents Na_V_ intensity at .5‐μm increments along the AIS. Black closed dots, WT; gray open squares, *Scn1a* ^*+/−*^ mice. Error bars represent 95% confidence intervals. Statistical significance: ***p < .001; n.s., not significant. (B) VGSC subunit quantification at P17–P19. Na_V_1.1 is localized along the proximal AIS and is decreased in *Scn1a* ^*+/−*^ compared to WT (genotype effect, *p* < .001; localization effect, *p* < .001; interaction of localization and genotype, *p* < .001; WT *n* = 19 cells from *n* = 3 mice; *Scn1a* ^*+/−*^ *n* = 26 cells from *n* = 4 mice). Na_V_1.2 is not increased above background (genotype effect, *p* = .95; localization effect, *p* = .16; interaction, *p* = .59; WT *n* = 20 cells from *n* = 3 mice; *Scn1a* ^*+/−*^ *n* = 19 cells from *n* = 4 mice). Na_V_1.3 is not increased above background (genotype effect, *p* = .34; localization effect, *p* = .10; interaction, *p* = .27; WT *n* = 6 cells from *n* = 1 mouse; *Scn1a* ^*+/−*^ *n* = 11 cells from *n* = 3 mice). Na_V_1.6 is localized along the distal AIS in both WT and *Scn1a* ^*+/−*^ mice (genotype effect, *p* = .22; localization effect, *p* < .001; interaction, *p* = .99; WT *n* = 44 cells from *n* = 4 mice; *Scn1a* ^*+/−*^ *n* = 60 cells from *n* = 5 mice). (D) VGSC subunit quantification at P35–P37. Na_V_1.1 is localized along the proximal AIS and remains decreased in *Scn1a* ^*+/−*^ compared to WT (genotype effect, *p* < .001; localization effect, *p* < .001; interaction of localization and genotype, *p* < .001; WT *n* = 24 cells from *n* = 3 mice; *Scn1a* ^*+/−*^ *n* = 24 cells from *n* = 3 mice). Na_V_1.2 is not increased above background (genotype effect, *p* = .21; localization effect, *p* = .35; interaction, *p* = .29; WT *n* = 20 cells from *n* = 3 mice; *Scn1a* ^*+/−*^ *n* = 21 cells from *n* = 3 mice). Na_V_1.3 is not increased above background (genotype effect, *p* = .29; localization effect, *p* = .95; interaction, *p* = .99; WT *n* = 6 cells from *n* = 2 mice; *Scn1a* ^*+/−*^ *n* = 12 cells from *n* = 2 mice). Na_V_1.6 is distributed along the distal AIS, with a significant upregulation and proximal shift in *Scn1a* ^*+/−*^ compared to WT (genotype effect, *p* < .001; localization effect, *p* < .001; interaction, *p* = .015; WT *n* = 41 cells from *n* = 3 mice; *Scn1a* ^*+/−*^ *n* = 58 cells from *n* = 6 mice).

We performed immunohistochemistry for all brain‐expressed VGSC α subunits in neocortex from *Scn1a* ^*+/−*^ mice and age‐matched WT littermate controls across development. Two time points were investigated: (1) developing/nestling mice at P17–P19, coinciding with the period of reduced excitability in *Scn1a* ^*+/−*^ PV+INs; and (2) juvenile/young adult mice at P35–P37, when *Scn1a* ^*+/−*^ PV+IN firing frequency is recovered.^14^ VGSC localization remained qualitatively similar between time points in WT (Figure 1). Na_V_1.1 was detected in a compact region colocalizing with the AIS marker AnkG both at the PV+IN AIS and farther away from cell bodies, likely representing nodes of Ranvier and/or preterminal axons. Na_V_1.2 was expressed at AISs throughout the neuropil, but not at PV+IN AISs (Figures 1 and 2). Na_V_1.3 was expressed in a small subset of neocortical somata, including of PV+INs, but not at the AIS; such labeling appeared to be intracellular. Na_V_1.6 was broadly expressed at the distal AIS thoughout, including the PV+IN AIS.

VGSC levels were quantified along the PV+IN AIS of *Scn1a* ^*+/−*^ and WT at both time points (Figure 2). A linear mixed‐effects model for all conditions (genotype, age, localization, and VGSC subunit) revealed main effects for AIS localization (*p* < .01) and VGSC subunit (*p* < .001). Multiple significant interaction effects indicated that the influence of age and genotype varied by VGSC subunit. Therefore, follow‐up two‐way ANOVAs were conducted separately for each VGSC subunit to assess the interaction between VGSC subunit and the effects of age and genotype. Full statistical results are provided in the Tables S1–S4.

Na_V_1.1 signal was concentrated at the proximal portion of the AIS (nearest the soma) at both P17–P19 (*p* < .001) and P35–P37 (*p* < .001). Na_V_1.1 signal intensity was decreased in *Scn1a* ^*+/−*^ compared to WT both at P17–P19 (*p* < .001) and at P35–P37 (*p* < .001). In both genotypes, Na_V_1.6 distribution along the PV+IN AIS was enriched at the distal AIS at both P17–P19 (*p* < .001) and P35–P37 (*p* < .001). At P17–P19, there was no difference in Na_V_1.6 intensity or distribution between WT and *Scn1a* ^*+/−*^ PV+INs. However, at P35–P37, there was a marked increase in Na_V_1.6 intensity at the *Scn1a* ^*+/−*^ PV+IN AIS relative to age‐matched WT (*p* < .001), with a proximal shift of Na_V_1.6 distribution along the PV+IN AIS in *Scn1a* ^*+/−*^ mice (*p* = .015). In contrast, the only Na_V_1.2 and Na_V_1.3 signal detected along the AIS in WT or *Scn1a* ^*+/−*^ PV+INs at either time point was low‐intensity or background signal (Figures 1 and 2). These results demonstrate a selective and time‐dependent upregulation of Na_V_1.6 but not Na_V_1.1, 1.2, or 1.3 at the PV+IN AIS of *Scn1a* ^*+/−*^ mice relative to WT, apparent at the late juvenile/young adult time point, consistent with previous electrophysiological data.

## 4 DISCUSSION

At the PV+IN AIS, Na_V_1.1 localizes proximally and Na_V_1.6 distally. *Scn1a* haploinsufficiency decreases mouse PV+IN AP firing rates most prominently during early developmental stages (before P35); this firing pattern partially recovers by adulthood. Yet, the mechanism of this recovery remains unknown. We asked whether this recovery of fast‐spiking of PV+INs in *Scn1a* ^*+/−*^ mice could be due to upregulation of a CNS‐expressed VGSC subunit, either Na_V_1.1 itself, or Na_V_1.2, 1.3 and/or 1.6. Whereas Na_V_1.1 levels in *Scn1a* ^*+/−*^ PV+IN AISs are lower than in WT at P18 and remain lower at P35, Na_V_1.6 levels at the *Scn1a* ^*+/−*^ PV+IN distal AIS are selectively upregulated at P35+ (but not P17–P19), which corresponds to the observed time course of normalization of PV+IN fast‐spiking. This suggests that upregulation of Na_V_1.6 in *Scn1a* ^*+/−*^ PV+IN AISs may at least partially explain the functional recovery of fast‐spiking seen in the chronic phase of the disorder.

We did not find a qualitative switch in the VGSC subunit composition at the *Scn1a* ^*+/−*^ PV+IN AIS; Na_V_1.2 and Na_V_1.3 were not expressed in the PV+IN AIS of either genotype at either time point. Na_V_1.2 expression was noted at AISs throughout in the neuropil, likely corresponding to the AIS of pyramidal cells and non‐PV interneurons, but was not detected at the PV+IN AIS. Na_V_1.3 was sparsely expressed in neocortical cell bodies, including PV+INs, but with a pattern consistent with intracellular expression. Others have previously reported apparent upregulation of somatic Na_V_1.3 in hippocampus in *Scn1a* ^*+/−*^ mice.^5^, ^15^ We did not attempt to quantify this low‐level apparent intracellular Na_V_1.3 expression but did not detect Na_V_1.3 at the AIS or plasma membrane and did appreciate a qualitative genotype‐specific change by immunofluorescence.We did not assess potential ectopic expression/upregulation of non‐CNS expressed VGSCs.

This study does not prove that Na_V_1.6 upregulation is sufficient to rescue *Scn1a* ^*+/−*^ PV+IN firing per se. The PV+IN AIS is enriched in other ion channels and regulatory proteins, such as the potassium channel subunit K_V_3.1 (which contributes to fast‐spiking^16^) or VGSC β subunits.^17^ Shifts in Na_V_1.6 may be one component of a greater compensatory rearrangement, and further studies of the *Scn1a* ^*+/−*^ PV+IN AIS proteome could clarify this.

There is precedence for this observation, as similar compensatory shifts in AIS VGSC subunit composition have been described previously. In cells lacking Na_V_1.6, there is a cell type‐specific distal shift at the AIS in either Na_V_1.1 or Na_V_1.2 to sites normally occupied by Na_V_1.6.^18^ This suggests that VGSC subunits at the AIS can at least partially compensate for the absence of one another. The functional impact of substitution of Na_V_1.6 for Na_V_1.1 at the PV+IN AIS is unclear. There may be subtle biophysical differences between VGSC α subunits, and prior work suggests that VGSCs containing Na_V_1.6 subunits may exhibit a more hyperpolarized/negative *V*_1/2_ of activation and inactivation,^19^ although such properties in neurons are also regulated by expression of β subunits, phosphorylation and methylation state, and other factors. Another study demonstrated that it is instead the density of low‐threshold Na_V_1.6‐containing VGSCs that determines AP threshold in neocortical pyramidal cells.^20^ We previously found that the apparent voltage threshold (recorded at the soma) for AP generation in PV+INs in *Scn1a* ^*+/−*^ mice is lower at both P18–P21 and P35–P56 relative to age‐matched WT littermate controls,^7^ which is difficult to explain, but may be due to differences in input resistance, changes in the composition of AIS VGSCs, and/or shifts in the location of the spike‐generating zone. The lower AP threshold in PV+INs from *Scn1a* ^*+/−*^ mice relative to WT at the later (P35–P56) time point is consistent with and could be explained by the upregulation of Na_V_1.6 at the PV+IN axon observed here. However, we also found a lower apparent AP threshold in PV+INs from *Scn1a* ^*+/−*^ mice at P18–P21 yet found normal/unchanged expression of Na_V_1.6 at this early time point. Direct axonal recordings from PV+INs from *Scn1a* ^*+/−*^ versus WT mice across developmental time points and improved VGSC subunit‐specific pharmacology could clarify this issue.

Yet, the mechanism through which Na_V_1.6 is upregulated in *Scn1a* ^*+/−*^ PV+INs is unknown. This is an important issue, as identification of such a mechanism could potentially be targeted and manipulated therapeutically. Na_V_1.1 may restrict Na_V_1.6 from the proximal AIS through protein–protein interactions; loss of Na_V_1.1 could allow Na_V_1.6 to “fill in” and occupy open sites. Alternatively, PV+INs may detect or monitor intrinsic firing rates via an unknown mechanism and upregulate Na_V_1.6 in *Scn1a* ^*+/−*^ to reachieve fast‐spiking. Finally, increased activity in *Scn1a* ^*+/−*^ brain may lead to non‐cell‐autonomous Na_V_1.6 upregulation in PV+INs as a mechanism to balance circuit hyperexcitability.

Whether Na_V_1.6 upregulation is protective or pathologic remains unclear. Our experiments carry inherent survival bias; *Scn1a* ^*+/−*^ mice develop seizures and increased mortality beginning at ~P18, mirroring the time course of the rate of SUDEP‐like events. We only analyzed *Scn1a* ^*+/−*^ survivors here. It is thus unknown whether deceased mice failed to upregulate Na_V_1.6 (suggesting a protective role), Na_V_1.6 was upregulated to a greater extent in survivors, or Na_V_1.6 levels at PV+IN AISs are unrelated to survival. One approach to address this bias is the “mini‐slice” technique, in which a brain biopsy is removed for acute brain slice physiology at an early time point, facilitating subsequent assessment of the same mouse again at a later time point(s). Using this method, we previously showed that PV+INs from developing/nestling *Scn1a* ^*+/−*^ mice exhibit similar AP firing deficits across animals irrespective of whether these mice survived to young adulthood or were deceased from SUDEP. However, we observed that synaptic failures persist in mature survivors despite recovery of AP generation. Taken together, such observations suggest that future DS therapies may need to correct these downstream synaptic deficits. However, that symptom onset in *Scn1a* ^*+/−*^ mice occurs at/around P18 and that the complement of VGSC expression in PV+INs at this time point resembles WT suggest that the observed upregulation of AIS Na_V_1.6 in *Scn1a* ^*+/−*^ PV+INs at P35 is a compensatory response that is likely functionally related to partial recovery of AP generation and high‐frequency firing only and does not impact the fidelity of synaptic transmission.

In contrast, preclinical data show that Na_V_1.6 knockdown reduces seizure in *Scn1a* ^*+/−*^ models, suggesting that Na_V_1.6 upregulation may instead be pathological. However, the antiseizure effects of anti‐*Scn8a* antisense oligonucleotides in *Scn1a* ^*+/−*^ mice^21^ may instead be via nonspecific decrease of Na_V_1.6 in excitatory pyramidal cells, thereby blunting overall network excitation and seizures.

Antiseizure medications with a prominent mechanism of action of sodium channel blockade are considered to exacerbate seizures/epilepsy and be contraindicated in DS due to the already impaired sodium channel function of GABAergic inhibitory interneurons. However, some sodium channel blockers have been shown to be at least partially efficacious in mice^22^, ^23^ and humans^24^, ^25^ when administered beyond the early “febrile/onset” phase of the disorder (prior to P18 in mice and 4 years of age in humans). In the initial report of this phenomenon, all patients with DS younger than 4 years of age exhibited increased seizure frequency with lamotrigine therapy, whereas the four patients who exhibited no change or improvement in seizure frequency were age 4 years or older.^26^ We view these data as consistent with the possibility that any negative impact of sodium channel blockers in DS might be most relevant to the early febrile/onset phase, perhaps related to early impairment of PV+IN AP generation secondary to loss of Na_V_1.1. A “window” of early vulnerability may relate to a subsequent compensatory upregulation of Na_V_1.6. Further clarification of this issue may require more systematic investigation.

## 5 CONCLUSIONS

In summary, this study demonstrates how a perturbation in gene function interacts with brain development to engage a cascade that manifests as secondary neuronal changes to partially recover cell type‐specific neuronal excitability. As gene therapies advance toward the clinic, understanding the mechanisms of such downstream changes will be critical, and harnessing such endogenous mechanisms could itself be leveraged toward therapy.

## AUTHOR CONTRIBUTIONS

**Ania K. Dabrowski:** Methodology; investigation; visualization; writing—original draft; writing—review and editing. **Ala Somarowthu:** Software; formal analysis. **Sophie R. Liebergall**: Software; methodology. **Damaris N. Lorenzo:** Conceptualization. **Ethan M. Goldberg:** Conceptualization; resources; data curation; writing—review and editing; funding acquisition.

## FUNDING INFORMATION

This work was funded by NINDS F31NS132519 to S.R.L., NINDS R01NS110810 and NIMH R01MH136674 and R01MH127848 to D.N.L, and NINDS R01NS110869 and R01NS137604 to E.M.G.

## CONFLICT OF INTEREST STATEMENT

The authors declare no conflicts of interest.

## ETHICS STATEMENT

We confirm that we have read the Journal's position on issues involved in ethical publication and affirm that this report is consistent with those guidelines. The Children's Hospital of Philadelphia institutional animal care and use committee approved the experimental procedures used in this study (approval no. 1152) on January 16, 2024. All animal housing and experiments were conducted in strict accordance with the institutional guidelines for care and use of laboratory animals at Children's Hospital of Philadelphia.

## Supporting information


TABLE S1
TABLE S2
TABLE S3
TABLE S4

## Data Availability

The data that support the findings of this study are available from the corresponding author upon reasonable request.
