# Supplementary material for: Compensatory rearrangement of parvalbumin interneuron voltage‐gated sodium channel subunits in a mouse model of Dravet syndrome
Source: Epilepsia. 2026 Jun 8;67(8):e133–40. doi: 10.1002/epi.70336 (PMC13525587; doi:10.1002/epi.70336)
Supplement: Supplementary file 1 [file EPI-67-e133-s001.docx]

**Supplementary Tables**

**Supplementary Table 1.** Linear mixed-effects model results with localization along AIS, genotype, age, and NaV1.X subunit as fixed effects.

| Term | FStat | DF1 | DF2 | pValue |
| --- | --- | --- | --- | --- |
| (Intercept) | 27396.15656 | 1 | 1416.96802 | 0 |
| AIS localization | 1.75476309 | 38 | 11990.2655 | 0.002802786 |
| Age | 2.544724713 | 1 | 1416.96802 | 0.110886196 |
| Na_V_1.X subunit | 564.5453384 | 3 | 1720.63903 | 2.135E-255 |
| Genotype | 0.033491448 | 1 | 1416.96802 | 0.854818952 |
| AIS localization: Age | 0.812917935 | 38 | 11990.2655 | 0.786410081 |
| AIS localization: Na_V_1.X subunit | 51.84150107 | 114 | 11528.0388 | 0 |
| Age: Na_V_1.X subunit | 56.07916821 | 3 | 1720.63903 | 1.3886E-34 |
| AIS localization: Genotype | 2.03862733 | 38 | 11990.2655 | 0.000169598 |
| Age: Genotype | 9.427017395 | 1 | 1416.96802 | 0.002178768 |
| Na_V_1.X subunit: Genotype | 65.06346081 | 3 | 1720.63903 | 7.54471E-40 |
| AIS localization: Age: Na_V_1.X subunit | 1.497916724 | 114 | 11528.0388 | 0.00049836 |
| AIS localization: Age: Genotype | 0.434951286 | 38 | 11990.2655 | 0.999046583 |
| AIS localization: Na_V_1.X subunit: Genotype | 2.279443053 | 114 | 11528.0388 | 3.41413E-13 |
| Age: Na_V_1.X subunit: Genotype | 4.79703725 | 3 | 1720.63903 | 0.002482379 |
| AIS localization: Age: Na_V_1.X subunit: Genotype | 0.66246952 | 114 | 11528.0388 | 0.99783747 |

**Supplementary table 2.** 2-way ANOVA with genotype and ais as fixed effects.

| **P17-19** | | | | | **P35-37** | | | | |
| --- | --- | --- | --- | --- | --- | --- | --- | --- | --- |
| **NaV1.1** | | | | | **NaV1.1** | | | | |
| Term | FStat | DF1 | DF2 | pValue | Term | FStat | DF1 | DF2 | pValue |
| (Intercept) | 4400.31969 | 1 | 80.1583492 | 8.2997E-72 | (Intercept) | 7708.39834 | 1 | 84.0870338 | 1.7507E-84 |
| Genotype | 35.4387337 | 1 | 80.1583492 | 6.6311E-08 | Genotype | 49.7943156 | 1 | 84.0870339 | 4.4926E-10 |
| Localization | 37.3557268 | 39 | 1075.81278 | 5.885E-171 | Localization | 32.4938839 | 39 | 1127.55366 | 1.144E-155 |
| Genotype: Localization | 4.50945755 | 39 | 1075.81278 | 1.5172E-17 | Genotype: Localization | 12.2262317 | 39 | 1127.55366 | 3.3352E-62 |
| **NaV1.2** | | | | | **NaV1.2** | | | | |
| Term | FStat | DF1 | DF2 | pValue | Term | FStat | DF1 | DF2 | pValue |
| (Intercept) | 11870.7555 | 1 | 72.1577343 | 8.3137E-82 | (Intercept) | 8190.36173 | 1 | 64.1523666 | 2.159E-69 |
| Genotype | 0.00380708 | 1 | 72.1577343 | 0.95097113 | Genotype | 1.62818541 | 1 | 64.1523666 | 0.20655481 |
| Localization | 1.23392197 | 39 | 938.601329 | 0.1564934 | Localization | 1.07182961 | 39 | 922.215099 | 0.35434119 |
| Genotype: Localization | 0.9338362 | 39 | 938.601329 | 0.58769179 | Genotype: Localization | 1.1156186 | 39 | 922.215099 | 0.29070234 |
| **NaV1.3** | | | | | **NaV1.3** | | | | |
| Term | FStat | DF1 | DF2 | pValue | Term | FStat | DF1 | DF2 | pValue |
| (Intercept) | 4534.79027 | 1 | 29.9999532 | 2.6709E-34 | (Intercept) | 8025.93106 | 1 | 21.8083161 | 1.7245E-29 |
| Genotype | 0.94082343 | 1 | 29.9999532 | 0.33982188 | Genotype | 1.15715394 | 1 | 21.8083161 | 0.29381382 |
| Localization | 1.32285126 | 39 | 382.058589 | 0.09947388 | Localization | 0.64698324 | 39 | 333.455641 | 0.95067747 |
| Genotype: Localization | 1.13764276 | 39 | 382.058589 | 0.26911679 | Genotype: Localization | 0.54933253 | 39 | 333.455641 | 0.98766409 |
| **NaV1.6** | | | | | **NaV1.6** | | | | |
| Term | FStat | DF1 | DF2 | pValue | Term | FStat | DF1 | DF2 | pValue |
| (Intercept) | 11341.9698 | 1 | 356.421782 | 2.728E-272 | (Intercept) | 8157.04622 | 1 | 345.378194 | 2.444E-242 |
| Genotype | 1.52635914 | 1 | 356.421782 | 0.2174734 | Genotype | 55.6686862 | 1 | 345.378195 | 7.0189E-13 |
| Localization | 40.6235737 | 39 | 3166.6026 | 1.077E-245 | Localization | 46.7493972 | 39 | 2942.24678 | 2.56E-274 |
| Genotype: Localization | 0.50083205 | 39 | 3166.6026 | 0.99597089 | Genotype: Localization | 1.55838749 | 39 | 2942.24678 | 0.01510141 |

**Supplementary Table 3.** Post-hoc analysis of genotype differences across each Na_V_1.X subunit and age.

| Na_V_1.X subunit | Age | F | DF1 | DF2 | p | p_adj |
| --- | --- | --- | --- | --- | --- | --- |
| Na_V_1.1 | 18 | 35.98434499 | 1 | 81.1204838 | 5.29764E-08 | 8.47622E-07 |
| Na_V_1.1 | 35 | 52.05325566 | 1 | 88.0474182 | 1.7985E-10 | 2.87759E-09 |
| Na_V_1.2 | 18 | 0.00974466 | 1 | 72.1473727 | 0.921638283 | 1 |
| Na_V_1.2 | 35 | 1.439979739 | 1 | 69.1227043 | 0.234239373 | 1 |
| Na_V_1.3 | 18 | 0.947416151 | 1 | 19.9999963 | 0.342004103 | 1 |
| Na_V_1.3 | 35 | 0.931574978 | 1 | 24.8689354 | 0.343746672 | 1 |
| Na_V_1.6 | 18 | 1.674015976 | 1 | 355.70318 | 0.196560962 | 1 |
| Na_V_1.6 | 35 | 58.26589556 | 1 | 355.598973 | 2.12869E-13 | 3.40591E-12 |

**Supplemental Table 4.** Post hoc analysis of age differences across each Na_V_1.X subunit and genotype.

| Channel | Genotype | F | DF1 | DF2 | p | p_adj |
| --- | --- | --- | --- | --- | --- | --- |
| Na_V_1.1 | *Scn1a+/-* | 23.1745391 | 1 | 93.999754 | 5.63928E-06 | 9.02285E-05 |
| Na_V_1.1 | WT | 23.1006125 | 1 | 75.2118331 | 7.6861E-06 | 0.000122978 |
| Na_V_1.2 | *Scn1a+/-* | 12.77550808 | 1 | 69.1832481 | 0.000645351 | 0.01032562 |
| Na_V_1.2 | WT | 12.50880365 | 1 | 72.1046501 | 0.000712528 | 0.011400442 |
| Na_V_1.3 | *Scn1a+/-* | 0.726623621 | 1 | 26.8745576 | 0.401512858 | 1 |
| Na_V_1.3 | WT | 0.680981406 | 1 | 18.0105931 | 0.420045338 | 1 |
| Na_V_1.6 | *Scn1a+/-* | 105.4497822 | 1 | 428.087545 | 2.91402E-22 | 4.66243E-21 |
| Na_V_1.6 | WT | 14.48581213 | 1 | 259.328962 | 0.000176256 | 0.002820092 |
